# Supplementary material for: Structural order enhances charge carrier transport in self-assembled Au-nanoclusters
Source: Nat Commun. 2020 Dec 3;11:6188. doi: 10.1038/s41467-020-19461-x (PMC7713068; doi:10.1038/s41467-020-19461-x)
Supplement: Supplementary file 1 — Supplementary Information [file 41467_2020_19461_MOESM1_ESM.pdf]

# – Supplementary Information–

# Structural order enhances charge carrier transport in self-assembled Au-nanoclusters

Florian Fetzer[1#], Andre Maier[2,3#], Martin Hodas[4], Olympia Geladari[2,3], Kai Braun[2,3], Alfred J. Meixner[2,3], Frank Schreiber[3,4], Andreas Schnepf[1*], Marcus Scheele[2,3*]

1. Institut für Anorganische Chemie Universität Tübingen, Auf der Morgenstelle 18, D-72076 Tübingen, Germany
2. Institut für Physikalische und Theoretische Chemie, Universität Tübingen, Auf der Morgenstelle 18, D-72076 Tübingen, Germany
3. Center for Light-Matter Interaction, Sensors & Analytics LISA+, Universität Tübingen, Auf der Morgenstelle 15, D-72076 Tübingen, Germany
4. Institut für Angewandte Physik, Universität Tübingen, Auf der Morgenstelle 10, D-72076 Tübingen, Germany

[#] These authors contributed equally: Florian Fetzer, Andre Maier

[*] These authors jointly supervised this work: Andreas Schnepf (email: andreas.schnepf@uni-tuebingen.de), Marcus Scheele (email: marcus.scheele@uni-tuebingen.de)

## SEM imaging of micro-crystals

Figure S1a displays a scanning electron micrograph of a micro-crystal. Well-defined edges as well as an extremely flat surface are observed. Figures S1b-d show side-views of micro-crystals with different thicknesses. In side-view the sample is tilted by 85° with respect to the incoming electron beam. From this, the thickness of individual micro-crystals can be investigated.

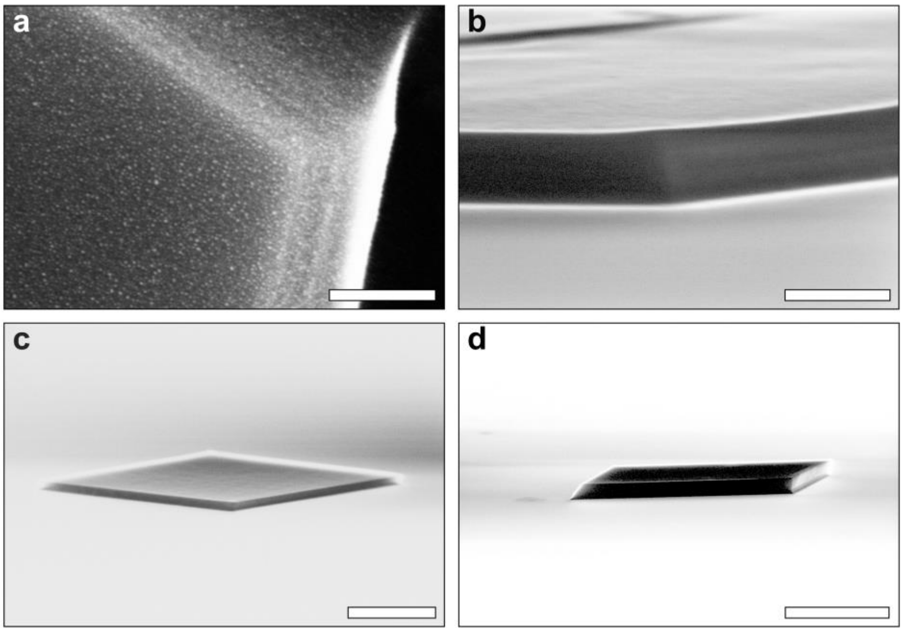


**Figure S1**: **SEM investigation of $Au_{32}$-NC micro-crystals.** (**a**) High-resolution SEM micrograph of an edge of a microcrystal. Individual NCs can be identified on the top-most layer as well as on the slightly tilted sidewall. However, structural arrangement of NCs cannot be resolved. Scale bar: 100 nm. (**b-d**) Side-view of different micro-crystals with thicknesses of ~200 nm (**b**), ~100 nm (**c**) and ~450 nm (**d**) under incident angle of 85°. Scale bars correspond to 300 nm (**b**), 1 µm (**c**) and 3 µm (**d**).

## Micro-crystal sample for GISAXS measurements

Figure S2 shows an optical micrograph of the micro-crystal sample used for GISAXS. The majority of micro-crystals is oriented flat on the surface. Minor agglomerates cause distortions of the resulting GISAXS pattern, as described in the main text.

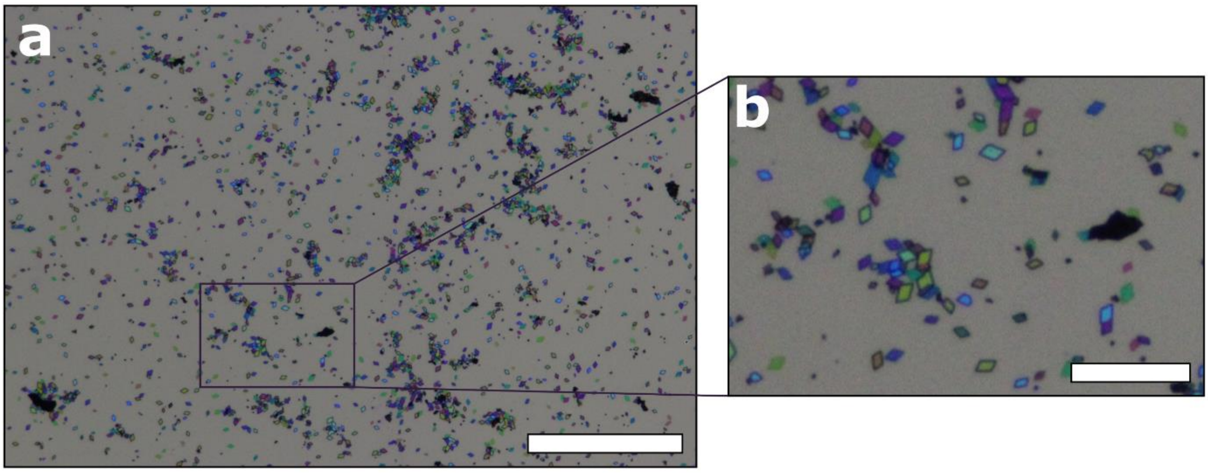


**Figure S2**: Optical micrograph of a $Si/SiO_x$ sample with an ensemble of hundreds of individual micro-crystals used for GISAXS measurements. Scale bars in (a) and (b) correspond to 200 µm and 50 µm, respectively.

## Dispersity of the micro-crystals

To determine the dispersity of the micro-crystals, scanning electron micrographs were used to measure the axis lengths of the micro-crystals (ImageJ software). The dispersity *Đ* is calculated according the IUPAC definition using the following equation

$$Đ = \frac{M_{\mathrm{w}}}{M_{\mathrm{n}}}, \qquad (S1)$$

where $M_{\mathrm{w}} = \frac{\Sigma_i N_i M_i^2}{\Sigma_i N_i M_I}$ and $M_{\mathrm{n}} = \frac{\Sigma_i N_i M_i}{\Sigma_i N_i}$. $N$ is given as the number of crystals with axis length $M$. Using this equation with measured axis lengths of ~100 micro-crystals yielded a dispersity of *Đ* = 1.07.

## Optical spectrum of $Au_{32}(^{n}Bu_3P)_{12}Cl_8$ nanoclusters

Figure S3 shows an optical absorbance spectrum of $Au_{32}$-NCs dissolved in hexane. Several distinct molecular-like transitions are observed, together with a HOMO-LUMO transition at 1.55 eV.

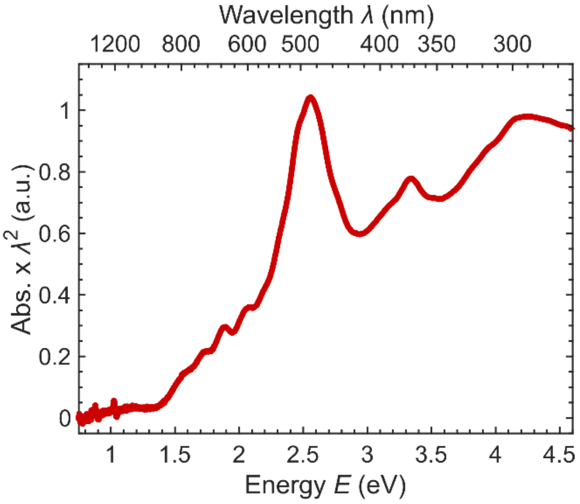


**Figure S3: Optical spectrum of $Au_{32}$-NCs**. Absorbance spectra of $Au_{32}$-NCs dispersed in hexane. Absorbance is energy corrected using the expression $I(E) = I(\lambda) \times \lambda^2$. The peak at 1.55 eV (800 nm) is attributed to the HOMO-LUMO transition. This spectrum corresponds to the spectrum shown in Figure 3a with larger energy range.

## Optical spectra of individual $Au_{32}$-NC micro-crystals

Figure S4 shows the absorbance spectra of several individual $Au_{32}$-NC micro-crystals, all exhibiting the enhanced peak at around 1.55 eV (800 nm).

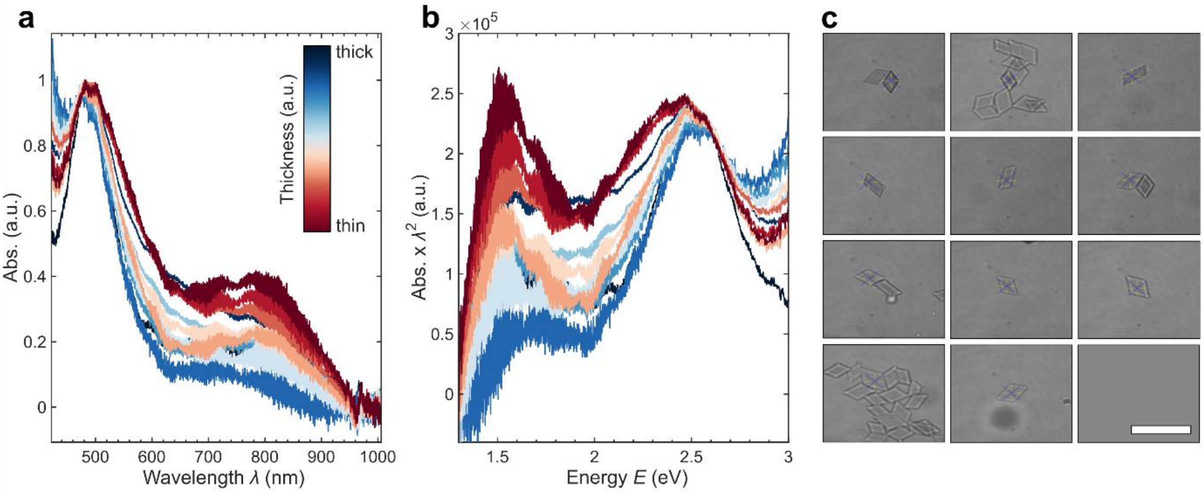


**Figure S4: Absorbance spectra of individual $Au_{32}$-NC micro-crystals**. (**a**) Absorbance as a function of wavelength $\lambda$ of eleven micro-crystals on glass. The legend indicates the qualitative thickness of the corresponding micro-crystals, determined via the grayscale values from the optical micrographs in (c). (**b**) The spectra from (a) with energy corrected absorbance (using the expression $I(E) = I(\lambda) \times \lambda^2$) as a function of energy. All micro-crystals exhibit the enhanced absorbance peak at around 1.55 eV (800 nm). All curves are normalized to the local maximum at 2.58 eV (480 nm). (**c**) Optical micrographs (camera images) of the eleven micro-crystals where absorbance was measured (labeled). From top left to bottom right, the grayscale value of the micro-crystals decreases, assuming the thickness to decrease as well. The scale bar corresponds to 40 µm and applies to all subfigures.

## Electrode layout for electronic measurements of individual micro-crystals

Optical micrographs of typical electrode devices (Si/$SiO_x$ substrate) are given in Figure S5. Adjacent electrodes form channels due to overlapping ends. The width of the overlap is 80 µm and the distance between electrodes defines the channel length *L*. On a single device, up to 330 individual channels are realized. By contacting the contact-pads of adjacent electrodes, every channel can be addressed individually. The devices are coated with micro-crystals as described in the Methods section. Micro-crystals which are bridging two adjacent electrodes can be contacted and probed individually. On a typical device, 10–40 individual micro-crystals can be investigated. Due to the relatively thin electrode thickness of ~10 nm, contacted micro-crystals are not free-standing but establish contact to the $SiO_x$ layer, manifested by the observed field-effect.

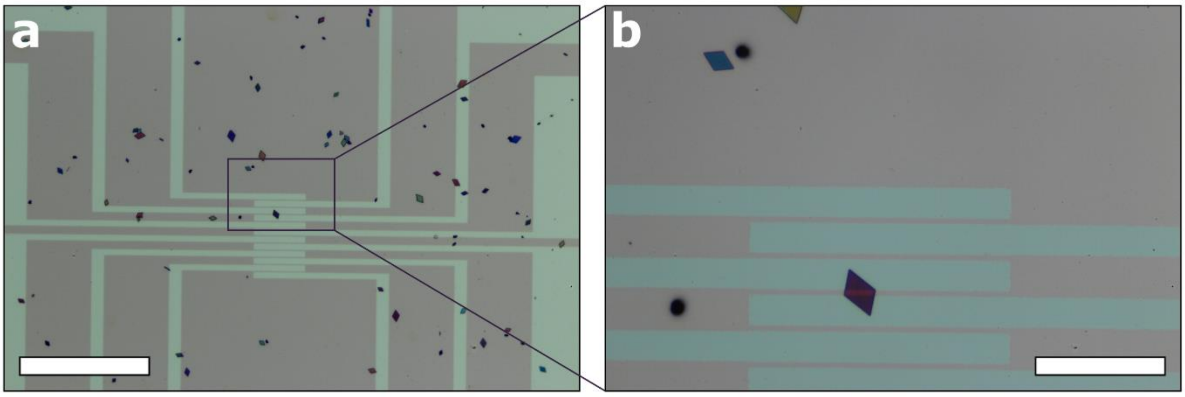


**Figure S5:** Optical micrographs of a typical electrode device with micro-crystals. Micro-crystals which are bridging two adjacent electrodes can be contacted and probed individually. (10 nm Au-electrodes on Si/$SiO_x$ wafer with 200 nm oxide thickness). Scale bars of (a) and (b) correspond to 200 µm and 40 µm, respectively.

## Evaluating the effective width of a micro-crystal within a channel

The effective width of a micro-crystal on a channel is described best by the mean of *W* along the channel. Figure S6 shows a SEM micrograph of a micro-crystal covering two channels. The measured conductance *G* of the channels is different, due to differences in effective width. For every channel, different widths are present (caused by the parallelogram shape). The calculated conductivities $\sigma$ of the two channels should be the same, since the same micro-crystal is probed. Normalizing the measured conductance *G* with the channel geometry $L/W$ gives essentially the same value (as thickness *h* is the same). This geometry normalized conductance values, using the mean width, are identical with 39.5 pS and 39.9 pS for the micro-crystals in the two different channels, respectively. Thus, using the mean width along the electric field is the most appropriate dimension.

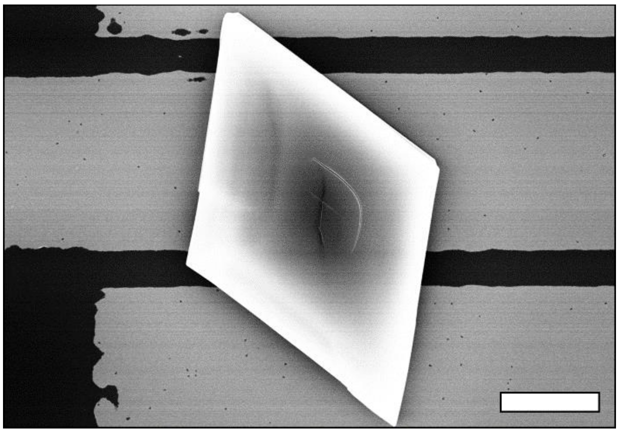

**Figure S6:** SEM micrograph of an individual micro-crystal, covering two different channels. Using the mean value of *W* to calculate the conductivity $\sigma$ yields the same value for geometry normalized conductance for both channels bridged by the same micro-crystal. Scale bar: 5 µm.

## *I*-*V* curves of $Au_{32}$-NC devices

Figure S7 shows representative *I*-*V* curves of a self-assembled $Au_{32}$-NC micro-crystal with conductivity $\sigma_{crystal} = 2.4 \times 10^{-4}$ S/m and a spin-coated $Au_{32}$-NC thin film with $\sigma_{film} = 4.4 \times 10^{-6}$ S/m.

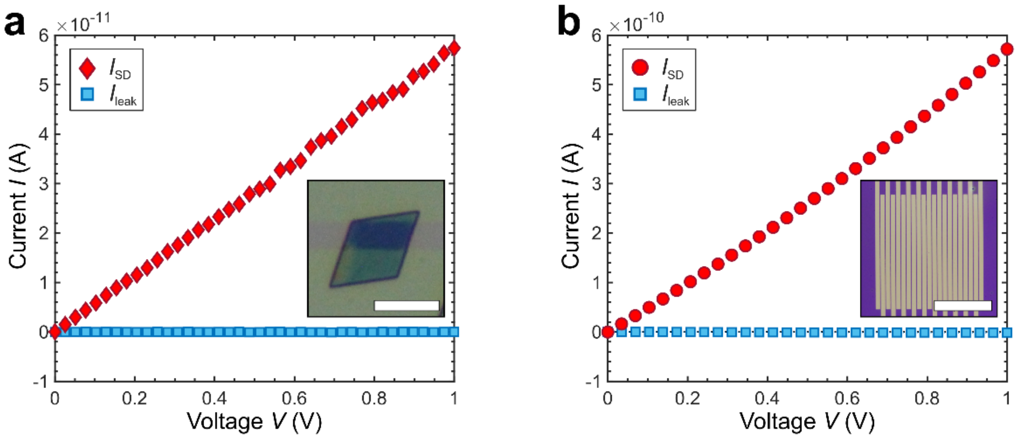


**Figure S7:** *I*-*V* curves of $Au_{32}$-NC devices. (**a**) Typical *I*-*V* curve of an individually probed micro-crystal. Fitting $I_{SD}$ (red) yields the conductance $G_{crystal}$ = 57 pS. The leakage (blue) is negligible. The inset displays the corresponding micro-crystal with $L$ = 2.8 µm, $W$ = 6.8 µm and $h$ = 98 nm. The conductivity can be calculated to $\sigma_{crystal} = 2.4 \times 10^{-4}$ S/m. Scale bar: 7 µm. (**b**) Typical *I*-*V* curve of $Au_{32}$-NC thin film. Fitting $I_{SD}$ (red) yields the conductance $G_{film}$ = 533 pS. The leakage (blue) is negligible. The inset displays the corresponding device with $L$ = 2.5 µm, $W$ = 1 cm and $h$ = 30 ± 2 nm. The conductivity can be calculated to $\sigma_{film} = 4.4 \times 10^{-6}$ S/m. Scale bar: 250 µm.

## Details on spin coated thin film samples

All thin film samples were prepared by spin coating and no post-coating techniques were applied. The samples were stored overnight to ensure full evaporation of residual solvents. Thin film samples for electronic measurements were placed under vacuum overnight in the probe station and measured under vacuum conditions (pressure of $\leq 10^{-5}$ mbar). Figure S8 displays optical and scanning electron micrographs of typical spin-coated $Au_{32}$-NC thin films.

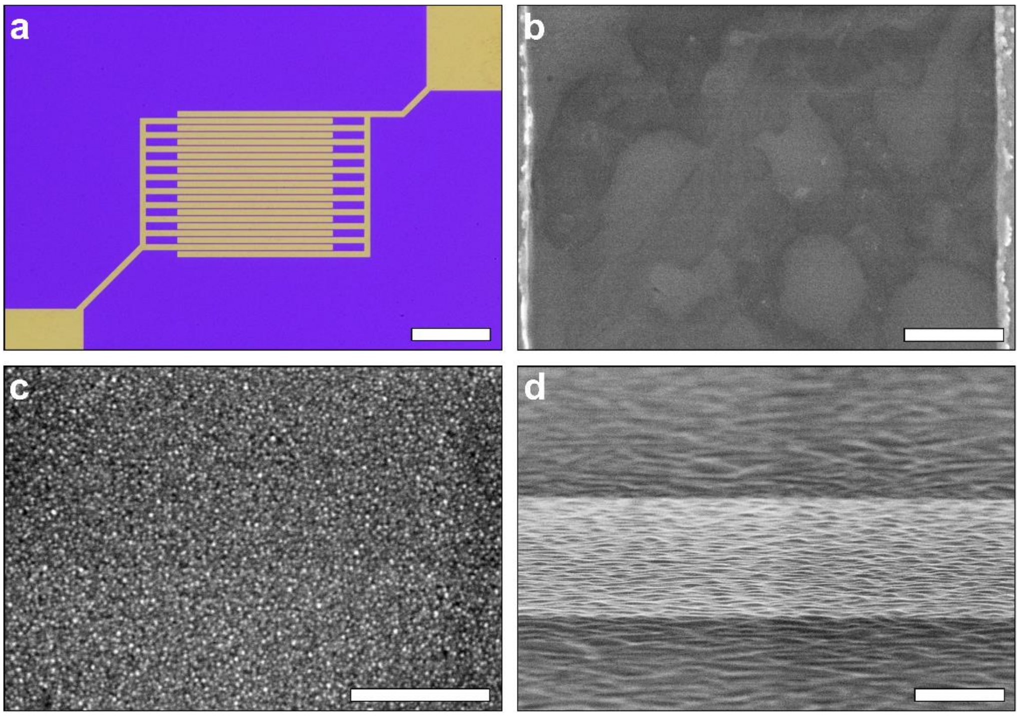


**Figure S8: Micrographs of spin-coated $Au_{32}$-NC thin films.** (**a**) Optical micrograph of a 30 ± 2 nm thin film on interdigitated Au electrodes of a $Si/SiO_x$ substrate. Optically, the thin film appears homogeneous over the entire displayed area of ~1.5 $mm^2$. Scale bar: 250 µm. (**b**) Scanning electron micrograph of the same film within a channel of $L$ = 2.5 µm (Au electrodes at left and right side). A continuous film with individual grains of 440 ± 130 nm length can be observed. Scale bar: 500 nm. (**c**) High-resolution SEM micrograph of the $Au_{32}$-NC thin film. Scale bar: 100 nm. (**d**) SEM micrograph under incident angle of 85° of a thin film within a channel of $L$ = 2.5 µm (Au electrodes in dark, channel gap in bright). A smooth and uniform surface can be observed. Scale bar: 1.5 µm.

## Thickness characterization of $Au_{32}$-NC thin films

Figure S9 displays the thickness characterization of spin-coated $Au_{32}$-NC thin films by profilometry (Bruker, Dektak XT). The samples were prepared with a scratch (Figure S9a) to identify the absolute film thickness by scanning across (Figure S9b). Several height profiles were taken per sample to calculate the mean value and standard deviation of the film thickness. Measuring the thickness on several positions of the samples and calculating the mean value ± standard deviation yields values of 30 ± 2 nm and 47 ± 4 nm (relative deviations of 6.7–8.5%).

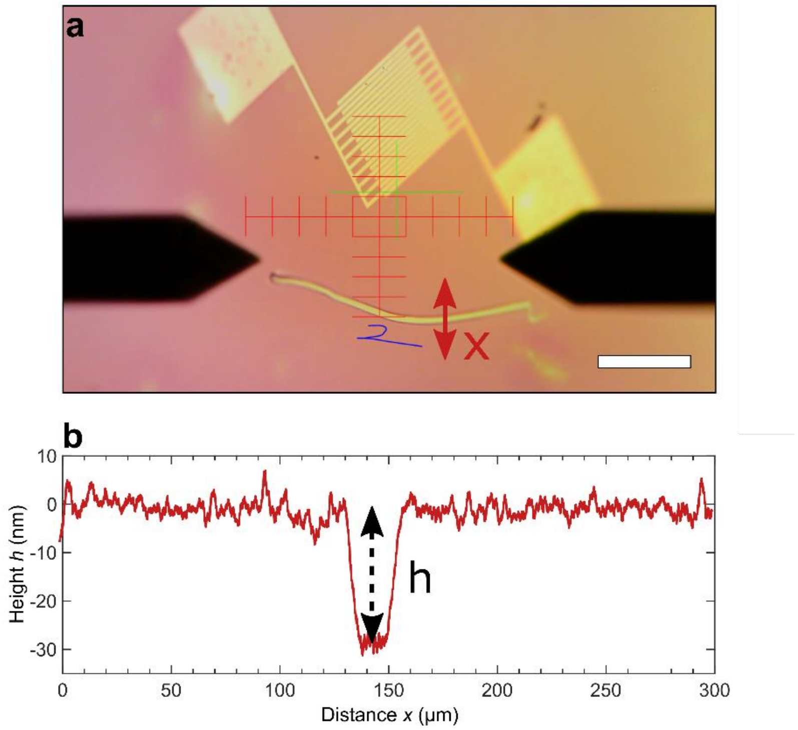


**Figure S9: Thickness characterization of $Au_{32}$-NC thin films by profilometry**. (**a**) Camera image of the profilometer showing an electrode device with a spin-coated $Au_{32}$-NC thin film and the stylus (as well as its reflection). The red arrow indicates the scanning direction *x* across a scratch within the thin film. Scale bar: 500 µm. (**b**) Corresponding height profile of the thin film revealing a thickness of $h$ = 30 nm and a root-mean-square roughness of 2.2 nm.

## Temperature dependent conductivity measurements

Figure S10a and S10c display typical plots of temperature-dependent conductivity of an $Au_{32}$-NC micro-crystal and a spin-coated thin film, respectively. Figure S10b and S10d show the corresponding Arrhenius plot, where ln($\sigma$) is plotted as a function of $T^{-1}$. Fitting the linear curve yields the activation energy $E_A$.

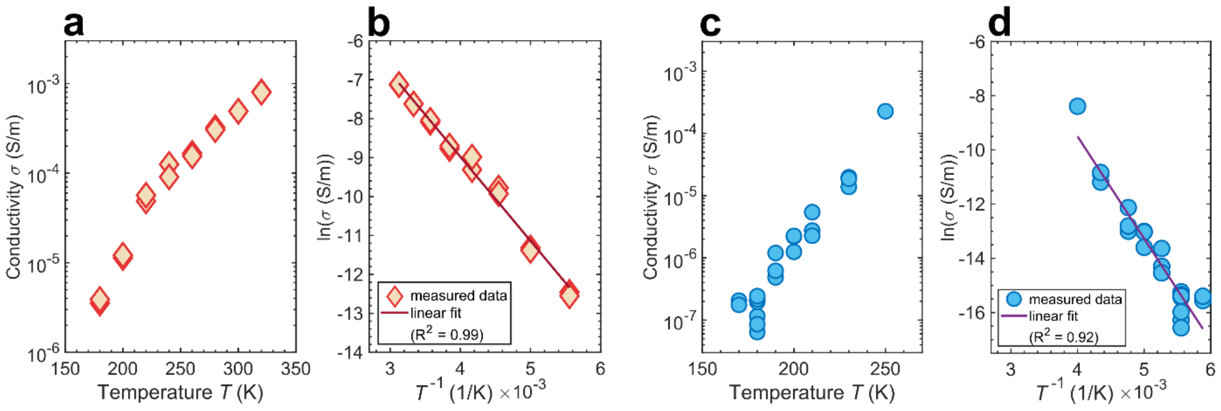


**Figure S10:** Temperature-dependent conductivity measurements of an $Au_{32}$-NC micro-crystal (a,b) and a spin-coated thin film (c,d). (**a**) Conductivity as a function of temperature of an individual micro-crystal with $L = 2.8$ µm, $W = 7.9 \pm 04$ µm and $h = 120$ nm. At every temperature step, several measurements were performed. This Figure corresponds to Figure 4d. (**b**) Arrhenius-plot of the data points shown in (b). The linear fit yields the activation energy $E_A \approx 0.2$ eV. The $R^2$ value of 0.99 indicates the goodness of the linear fitting. (**c**) Conductivity as a function of temperature of a thin film channel with $L = 2.5$ µm, $W = 1$ cm and $h = 30 \pm 2$ nm. At every temperature step, several measurements were performed. (**d**) Corresponding Arrhenius-plot of (c). The linear fit yields the activation energy $E_A \approx 0.33$ eV. The $R^2$ value of 0.92 indicates the goodness of the linear fitting. In the manuscript, we report the mean value of $E_A$ for micro-crystals and thin films, including the standard deviation, based on the evaluation of 25 and 7 of such plots, respectively. This yields $E_A = 227 \pm 17$ meV for micro-crystals and $E_A = 366 \pm 62$ meV for the thin films.

## Equations used for the investigation of electronic properties

The field-effect mobilities $\mu$ of individual micro-crystals or polycrystalline thin films are calculated using the gradual channel approximation, given in Equation S2. The charge carrier concentration $n$ is calculated using Equation S3.

$$\mu = \frac{\partial I_{\mathrm{SD}}}{\partial V_{\mathrm{G}}} \frac{L}{W} \frac{t_{\mathrm{ox}}}{\varepsilon_0 \varepsilon_{\mathrm{r}} V_{\mathrm{SD}}} \qquad \text{(S2)}$$

$$n = \frac{\sigma}{e\,\mu} \qquad \text{(S3)}$$

Being $\frac{\partial I_{\mathrm{SD}}}{\partial V_{\mathrm{G}}}$ the derivation of $I_{\mathrm{SD}}$ in FET transfer curves, $V_{\mathrm{SD}}$ the source-drain voltage, $\varepsilon_0\varepsilon_{\mathrm{r}}$ and $t_{\mathrm{ox}}$ the permittivity and the thickness (230 nm for interdigitated electrodes, 200 nm for micro-crystal devices) of the dielectric $SiO_x$ layer, respectively, and $e$ the elementary charge.

## Field-effect mobility of individual $Au_{32}$-NC microcrystals

Figure S11 shows the distribution of the field-effect hole mobility $\mu(h^+)$ of individual micro-crystals.

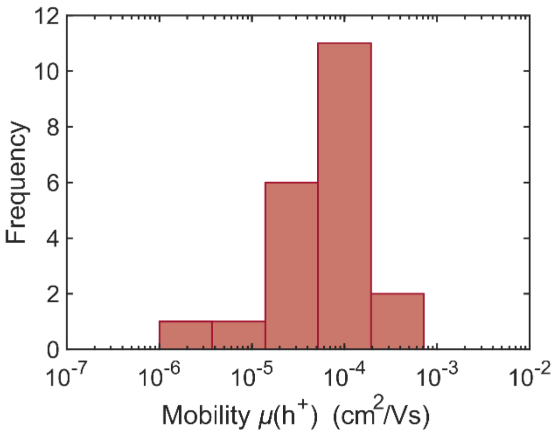


**Figure S11:** Distribution of the field-effect hole mobility $\mu(h^+)$ of 21 individual micro-crystals. All micro-crystals show p-type behavior. The mean value and standard deviation can be calculated to be $\mu(h^+) = 0.8 \times 10^{-4} \pm 0.58 \times 10^{-4}$ cm$^2$ V$^{-1}$ s$^{-1}$. Values up to $2 \times 10^{-4}$ cm$^2$ V$^{-1}$ s$^{-1}$ are observed.

A schematic drawing of the devices is given in Figure S12. The micro-crystals are deposited onto the electrodes with a thickness of ~10 nm. Accordingly, there is a gap between the micro-crystals and the dielectric of 0–10 nm. This is what we refer to as non-ideal channel geometry. In contrast, the spin-coated films form a relatively conformal layer within the channel, which leads to much better contact. Nonetheless, there is still an appreciable transconductance in the micro-crystals, which - after renormalization for the different channel geometry - is ~30 higher for the micro-crystals compared to the spincoated films. This further indicates the more efficient charge transport in highly ordered micro-crystals.

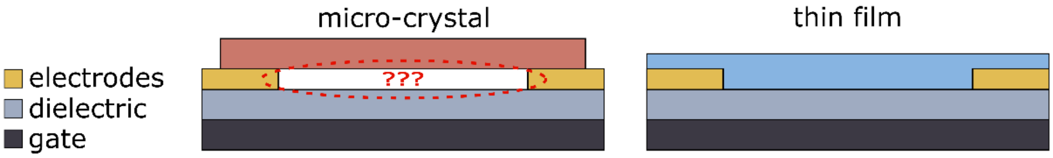


**Figure S12:** Schematic drawing of FET devices of a micro-crystal (left) and a thin film (right).

Details about the current normalization considering the different channel geometries:

- thin film: $I_{\text{film}}(-40\ V_G, 5\ V_{SD}) = 5.5 \times 10^{-8}$ A
- micro-crystal: $I_{\text{crystal}}(-40\ V_G, 5\ V_{SD}) = 9.0 \times 10^{-9}$ A

Normalized to geometry:

- thin film: $$\frac{I_{\text{film}} \bullet L}{W \bullet h} = \frac{5.5 \times 10^{-8}\ \text{A} \bullet 2.5 \times 10^{-6}\ \text{m}}{0.01\ \text{m} \bullet 30 \times 10^{-9}\ \text{m}} = 4.58 \times 10^{-4}\ \text{A/m}$$
- micro-crystal: $$\frac{I_{\text{crystal}} \bullet L}{W \bullet h} = \frac{9.0 \times 10^{-9}\ \text{A} \bullet 1.5 \times 10^{-6}\ \text{m}}{10 \times 10^{-6}\ \text{m} \bullet 100 \times 10^{-9}\ \text{m}} = 1.35 \times 10^{-2}\ \text{A/m}$$

Ratio: $$\frac{1.35 \times 10^{-2}\ \text{A/m}}{4.58 \times 10^{-4}\ \text{A/m}} = 29.5$$

## Evaluation of the contact resistance of $Au_{32}$-NC devices

To measure the contact resistance $R_C$ of the $Au_{32}$-NC devices, we apply the $Y$-function method (YFM), which is a common technique for the evaluation of MOSFETs and OFETs.[1–3]

Figure S13 illustrates the YFM technique to estimate the $R_C$ of a FET device (exemplarily shown for an $Au_{32}$-NC thin film channel).

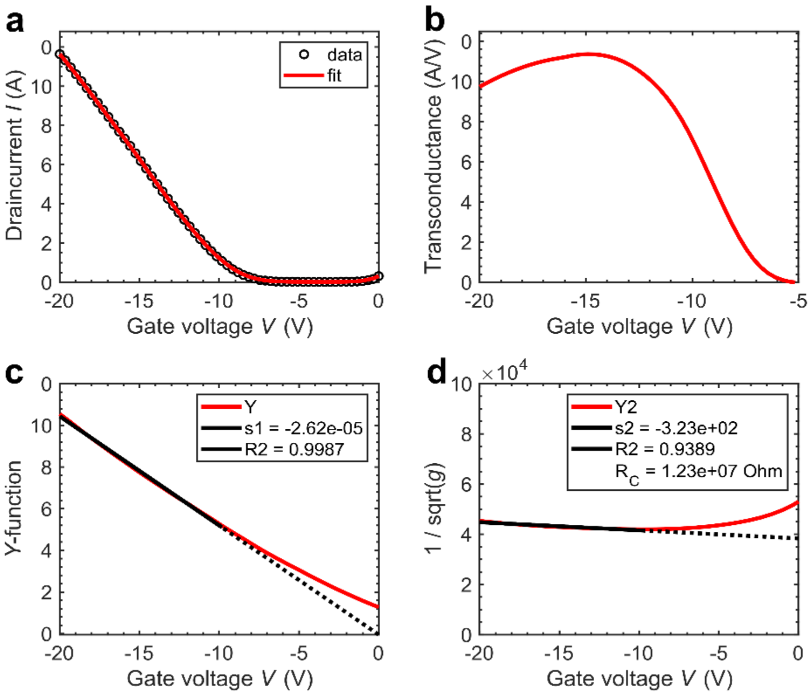


**Figure S13:** $Y$-function method (YFM) to estimate the contact resistance $R_C$ of an individual FET device. (**a**) Transfer characteristic of an $Au_{32}$-NC thin film p-type FET at $V_{SD}$ = 1 V ($L$ = 2.5 µm, $W$ = 1 cm). (**b**) Transconductance versus gate voltage $V_G$. (**c**) $Y$-function versus $V_G$. Fitting the linear regime yields the slope $s1$. (**d**) $\frac{1}{\sqrt{g}}$ versus $V_G$. Fitting the linear regime yields the slope $s2$. The $R^2$ values in (c) and (d) verify the goodness of the linear fit. $R_C$ is calculated to $1.2 \times 10^7\ \Omega$. The total resistance of this device is $1.9 \times 10^9\ \Omega$.

From the transfer characteristic $I_{SD}$ vs. $V_G$ (Figure S13a) the transconductance $g$ is determined as $g = \partial I_{SD} / \partial V_G$ (Figure S13b). The $Y$-function is defined in Equation S4 as

$$Y = \frac{I_{SD}}{\sqrt{g}} \qquad \text{(S4)}$$

and fitting $Y$ as a function of $V_G$ in the linear regime yields the slope $s1$ (Figure S13c). Next, the function $\frac{1}{\sqrt{g}}$ versus $V_G$ is determined and linearly fitted to calculate the slope $s2$ (Figure 13d). The contact resistance $R_C$ is calculated using Equation S5 as

$$R_C = V_{SD} \times \frac{s2}{s1} \qquad (S5)$$

Using this method, we determine the contact resistance of thin films and microcrystal channels as $R_{C,films} \approx 1–3 \times 10^7\ \Omega$ and $R_{C,crystals} \approx 2 \times 10^8\ \Omega$, respectively. In contrast, the total resistances are $R_{films} \approx 0.5–2 \times 10^9\ \Omega$ and $R_{crystals} \approx 2–20 \times 10^9\ \Omega$. Thus, the contact resistances are only ~2% and ~1–10% for thin film and microcrystal devices, respectively.

Hence, the $Y$-function method verifies the applicability of simple 2-point-probe measurements, as the effect of $R_C$ is negligible.[1–3]

## Calculation of the Coulomb charging energy $E_C$

The estimation of the Coulomb charging energy is performed as described below.

The Coulomb charging energy $E_C$ is given in Equation S6.

$$E_C = \frac{e^2}{2\,C_\Sigma} \qquad (S6)$$

Here, $e$ is the elementary charge and $C_\Sigma$ the total capacitance of the particle to its surrounding. The interparticle capacitance can be estimated using Equation S7.[4,5]

$$C \approx 2\,\pi\,\varepsilon_0 \varepsilon_r r \ln\left(\frac{r+d}{d}\right) \qquad (S7)$$

Here, $\varepsilon_0$ is the vacuum permittivity, $\varepsilon_r$ the dielectric constant of the surrounding medium (~2.0–2.5 for alkanes and phosphine), $r$ is the NC radius (~0.45 nm) and $2d$ the interparticle distance. Knowing that individual micro-crystals are oriented face-on to the surface, the in-plane Au core-core distance corresponds to the axis $a$ and $b$ with ~1.9 nm. As the core size is 0.9 nm the

interparticle distance is $2d \approx 1.0$ nm. As each NC in the array has eight nearest neighbours, the total capacitance can be calculated to $C_\Sigma = 8\ C$. Accordingly, an estimation of charging energy yields $E_C \approx 276$ meV.

## Details on self-assembly process of $Au_{32}$-NC micro-crystals

Different parameters have been investigated to tune the morphology and amount of the micro-crystals. Using a larger quantity of particle solution leads to a higher amount of micro-crystals on the substrate (Figure S14). The same effect can be achieved by increasing the preparation time which allows more crystals to trickle through the subphase. Empirically, 45 min and a volume of 200 µl have shown best results in terms of crystal density along with reasonable preparation times. The influence of the chosen solvent is the following: $Au_{32}$-NC dispersions with hexane yield thicker micro-crystals (up to several hundreds of nanometers), whereas octane lead to the formation of thinner micro-crystals (thicknesses of 50–100 nm). For heptane, intermediate thicknesses can be achieved.

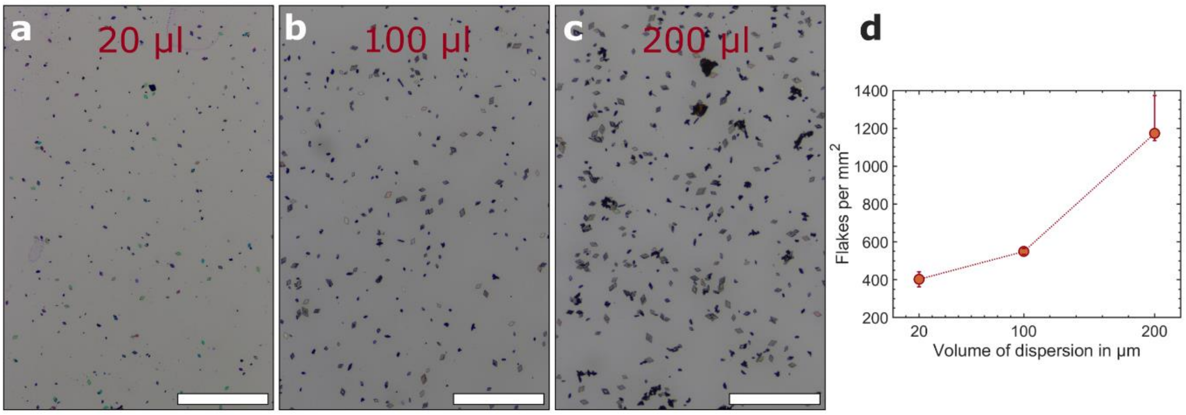


**Figure S14:** Effect of dispersion volume on self-assembly process. At a fixed dispersion solvent (heptane) and concentration (0.5 mM), the number of micro-crystals can be varied. Dispersion volumes of (a) 20 µl, (b) 100 µl and (c) 200 µl were used. (d) Number of micro-crystals per mm$^2$ as a function of dispersion volume. Scale bars correspond to 200 µm.

## Mechanism of micro-crystal self-assembly

To investigate the place and process of the formation of the micro-crystals, a self-built interference reflection microscope was used. A Framos Lt 225 camera with 16 nm/px resolution was used along with a Nikon TIRF objective with oil immersion and a numerical aperture of NA = 1.52. The sample was illuminated by a Rebel High Power LED with a wavelength of 460 nm. A Teflon tube was sealed onto a glass cover slide, filled with acetone and placed onto the microscope. The microscope was focused just above the glass slide into the subphase. An $Au_{32}$-NC solution (0.5 mM, octane) was added onto the subphase to start the process of self-assembly. After the duration of 15 min, the sudden appearance of micro-crystals was observed. From this, we deduce that the process of crystallization does not take place at the substrate but at the liquid-air interface. From there, the micro-crystals start to sink down through the subphase as soon as they reach a critical mass. After reaching the glass slide/substrate, the micro-crystals are able to move laterally within the subphase along the bottom. Upon removal of the subphase, the micro-crystals are deposited onto the substrate.

## Supporting References

1. Ghibaudo, G. New method for the extraction of MOSFET parameters. *Electron. Lett.* **24,** 543–545 (1988).
2. Xu, Y., Minari, T., Tsukagoshi, K., Chroboczek, J. A. & Ghibaudo, G. Direct evaluation of low-field mobility and access resistance in pentacene field-effect transistors. *J. Appl. Phys.* **107,** 114507 (2010).
3. Liu, C., Xu, Y. & Noh, Y.-Y. Contact engineering in organic field-effect transistors. *Mater. Today* **18,** 79–96 (2015).
4. Zabet-Khosousi, A. & Dhirani, A.-A. Charge transport in nanoparticle assemblies. *Chem. Rev.* **108,** 4072–4124 (2008).
5. Black, C. T., Murray, C. B., Sandstrom, R. L. & Sun, S. Spin-dependent tunneling in self-assembled cobalt-nanocrystal superlattices. *Phys. Rev. B* **290,** 1131–1134 (2000).
